# Supplementary material for: Clonal evolutionary analysis reveals patterns of malignant transformation of Intraductal Papillary Mucinous Neoplasms of the pancreas
Source: Nat Commun. 2026 Mar 4;17:3427. doi: 10.1038/s41467-026-69762-w (PMC13076896; doi:10.1038/s41467-026-69762-w)
Supplement: Supplementary file 1 — Supplementary Information [file 41467_2026_69762_MOESM1_ESM.pdf]

Supplementary Figures

Figure S1A. CONSORT diagram illustrating sample collection and analysis, and clinical and histological characteristics as detailed in the Sample Manifest. IPMN and PDAC classification and subtyping for each collected sample were determined based on morphological evaluation using slides of fresh-frozen tissue. The table also highlights cases where IPMN and PDAC are present within the same sample

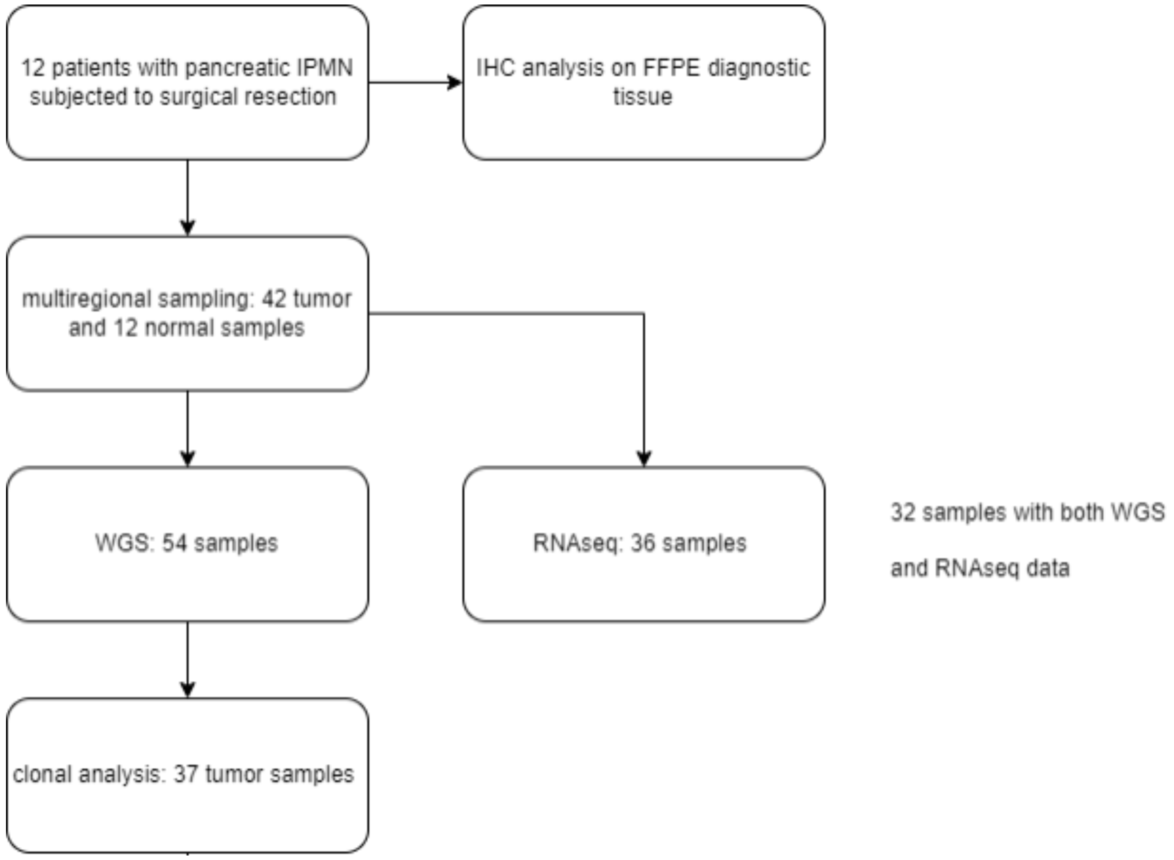

ID		Pathology						Clinical			Analysis		
ID samples	ID pathology	diagnosis	IPMN	IPMN histotype	IPMN grade of dysplas	Invasive carcinoma	PDAC histotype	Radiology	Sex	Age	WGS	Included in clon	RNA seq
case2_N		normal						head	m	73	x		
case2_S1	IPMN_HGD	IPMN	x	INT	HGD						x		x
case2_S10	IPMN_LGD	IPMN	x	GAS	LGD						x	x	x
case2_S2	IPMN_HGD	IPMN	x	INT	HGD						x	x	x
case2_S3	PDAC	PDAC + IPMN	x	INT	HGD	x	TUBULAR with COLLOID aspects				x		x
case2_S4	IPMN_HGD	IPMN	x	INT	HGD						x	x	x
case2_S9	IPMN_HGD	IPMN	x	INT	HGD						x		
case 3_N		normal						head	m	66	x		
case3_S1	IPMN_LGD	IPMN	x	PB	LGD						x	x	
case3_S2	IPMN_LGD	IPMN	x	PB	LGD						x	x	
case3_S4	IPMN_LGD	IPMN	x	PB	LGD						x	x	
case3_S5	PDAC	PDAC				x	TUBULAR				x		
case4_N		normal						head	m	58	x		
case4_S1	IPMN_LGD	IPMN	x	GAS	LGD						x	x	x
case4_S2	PDAC	PDAC + IPMN	x	GAS	LGD	x	TUBULAR				x	x	x
case4_S3	IPMN_LGD	IPMN	x	GAS	LGD						x	x	x
case4_S4	IPMN_LGD	IPMN	x	GAS	LGD						x	x	x
case4_S5	IPMN_LGD	IPMN	x	GAS	LGD						x	x	x
case6_N		normal						head	f	60	x		
case6_S7	IPMN_HGD_PDAC	IPMN with micorinvasive PDAC	x	PB	HGD	x	TUBULAR				x	x	x
case6_S8	PDAC	PDAC				x	TUBULAR				x	x	x
case6_S9	PDAC	PDAC				x	TUBULAR				x	x	
case7_N		normal						head /unc	f	65	x		
case7_S1	IPMN_HGD	IPMN	x	PB	HGD						x	x	x
case7_S2	PDAC	PDAC				x	TUBULAR						x
case7_S4	PDAC	PDAC + IPMN	x	PB	HGD	x	TUBULAR				x	x	x
case7_S5	IPMN_HGD_PDAC	IPMN with microinvasive PDAC	x	PB	HGD	x	TUBULAR				x	x	x
case9_N		normal						head	f	71	x		
case9_S2	PDAC	PDAC				x	TUBULAR				x	x	x
case9_S3	PDAC	PDAC				x	TUBULAR				x	x	x
case9_S4	IPMN_HGD_PDAC	IPMN with microinvasive PDAC	x	PB		x	TUBULAR				x	x	x
case9_S6	PDAC	PDAC				x	TUBULAR				x	x	
case10_N		normal						body/tail		62	x		
case10_S2	IPMN_LGD	IPMN	x	GAS (rare INT foci)	LGD				f		x	x	x
case10_S5	IPMN_LGD	IPMN	x	GAS (rare INT foci)	LGD						x	x	x
case11_N		normal						head/PU	m	54	x		
case11_S7	PDAC	PDAC		TUB		x					x		x
case11_S8	PDAC	PDAC		TUB		x					x		
case11_S9	PDAC	PDAC		TUB		x					x		
case12_N		normal						head	m	52	x		
case12_S1	IPMN_LGD	IPMN	x	INT	LGD						x	x	x
case12_S2	IPMN_LGD	IPMN	x	INT	LGD						x	x	x
case12_S3	IPMN_HGD	IPMN	x	INT	HGD						x	x	x
case12_S4	IPMN_LGD	IPMN	x	INT	LGD						x		x
case12_S5	IPMN_LGD	IPMN	x	INT	LGD						x	x	x
case13_N		normal						head	f	55	x		
case13_S3	PDAC	PDAC				x	COLLOID				x	x	x
case13_S4	cronic_pancreatitis	cronic pancreatitis		stromal component							x		x
case13_S5	PDAC	PDAC + IPMN	x	INT	HGD	x	COLLOID				x	x	x
case15_N		normal						body/tail	m	77	x		
case15_S1	IPMN_LGD	IPMN	x	GAS / PB	LGD						x	x	x
case15_S10	PDAC	PDAC		TUBULAR		x	TUBULAR				x	x	x
case15_S11	PDAC	PDAC		TUBULAR		x	TUBULAR				x	x	x
case15_S3	IPMN_LGD	IPMN	x	PB	LGD						x	x	x
case15_S4	IPMN_HGD	IPMN	x	PB	HGD						x	x	x
case16_N		normal						head	m	82	x		
case16_S2	IPMN_HGD	cystic lesion	x	INT	HGD						x	x	x
case16_S4	PDAC	PDAC				x	COLLOID				x	x	x
case16_S5	PDAC	PDAC				x	COLLOID				x		

Figure S1A2. Pipelines for WGS analysis

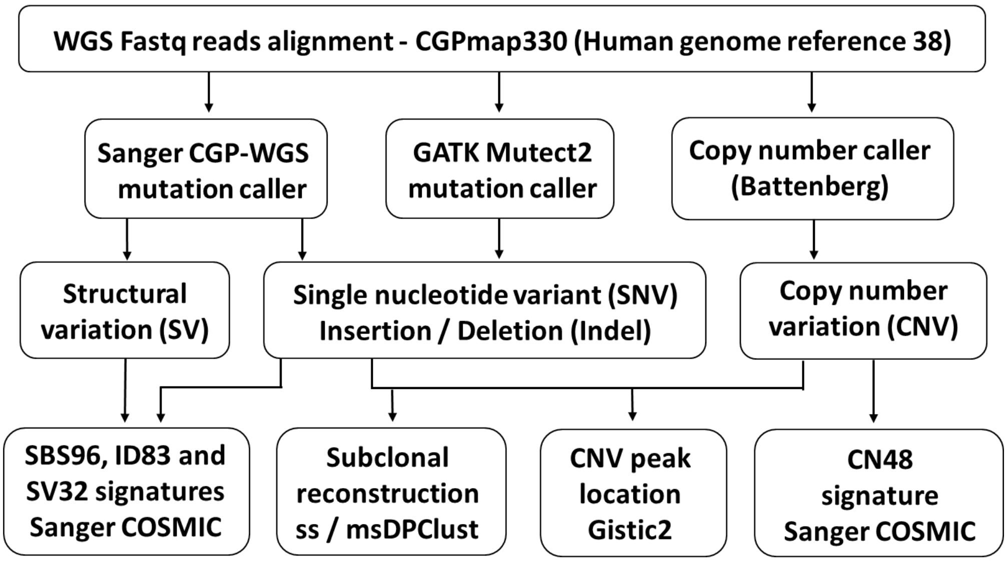

Figure S1B1 Comparison of Somatic Alterations Between IPMNs (n=22) and IPMN-Derived PDACs (n=19). (a) Tumor mutation burden (TMB) (p = 0.0001), (b) the number of SNVs and indels across genomic regions, and (c) structural variant (SV) types were compared between tumour groups. Significant differences were assessed using a two-sided Fisher–Pitman permutation test. Box plots display the median (centre line), the interquartile range (box), and the minima and maxima (whiskers). Source data are provided as a Source Data file.

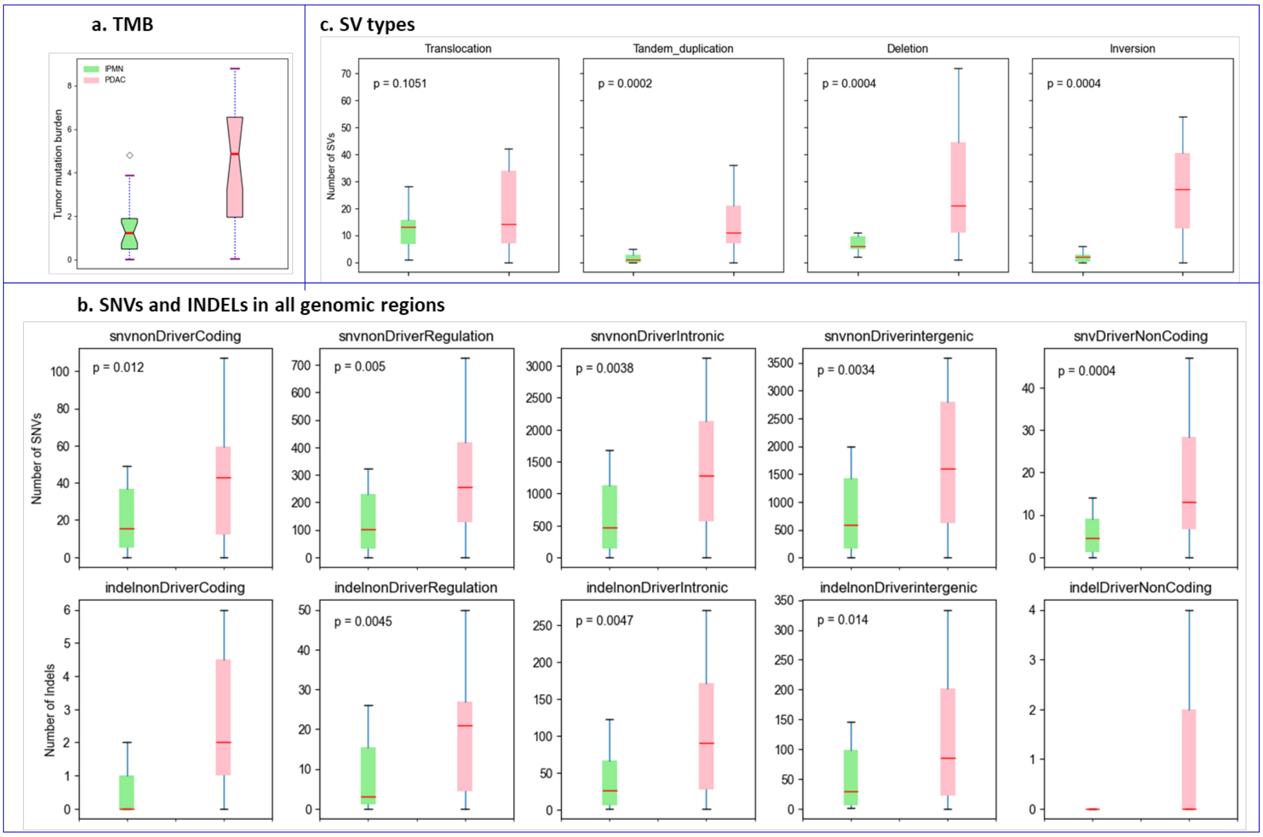

Figure S1B2. Analysis of CCF and VAF in KRAS missense IPMN and IPMN-Derived PDACs. Statistical analysis (Fisher–Pitman permutation test) showed no significant differences in the cancer cell fractions (CCFs) of KRAS between IPMN and IPMN-derived PDAC. Source data are provided as a Source Data file.

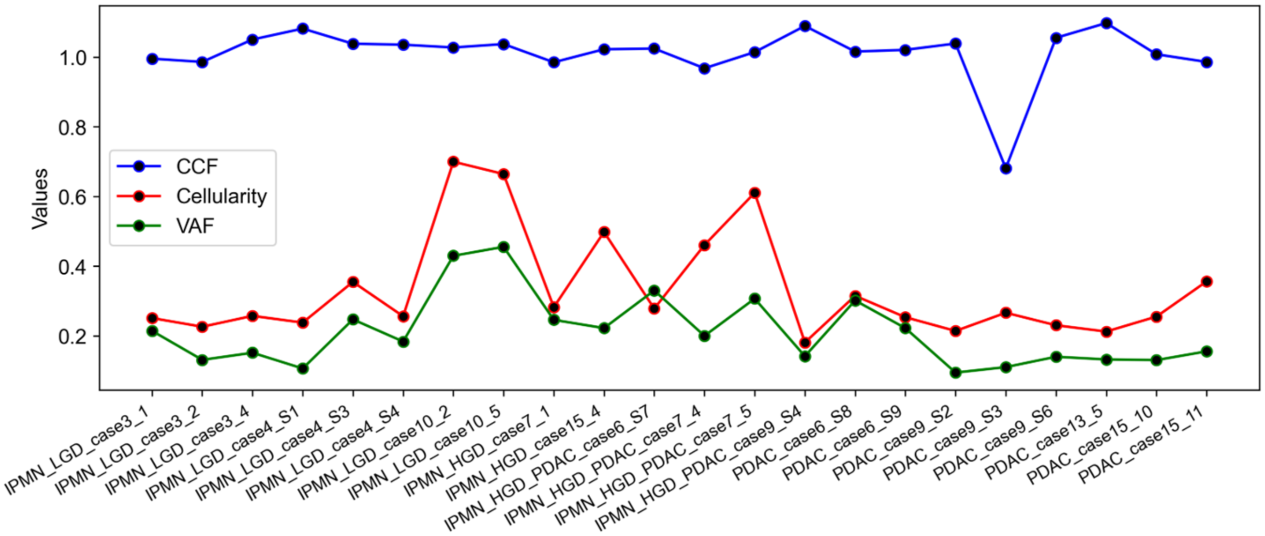

Figure S1B3. KRAS amino acid substitution (missense). The most frequently mutated gene, KRAS, exhibited hotspot mutations (15/22 G12D, 5/22 G12V, 2/22 G12R) in both IPMN and PDAC samples.

G12D	G12V	G12R
case15_S4_IPMN_LGD	case4_S1_IPMN	case15_S10_PDAC
case3_S1_IPMN_LGD	case7_S1_IPMN_HGD	case15_S11_PDAC
case3_S2_IPMN_LGD	case7_S5_IPMN_PDAC	
case3_S4 _IPMN_LGD	case7_S4_PDAC	
case10_S2_IPMN	case13_S5_PDAC	
case10_S5_IPMN		
case4_S3_IPMN		
case4_S4_IPMN		
case6_S7_IPMN_PDAC		
case9_S4_IPMN_PDAC		
case6_S8_PDAC		
case6_S9_PDAC		
case9_S3_PDAC		
case9_S6_PDAC		

Figure S1B4. Driver SNV / INDELs and CNV in IPMNs and IPMN-Derived PDACs (p < 0.05). Significant differences were tested using Fisher's exact test with FDR correction. Source data are provided as a Source Data file.

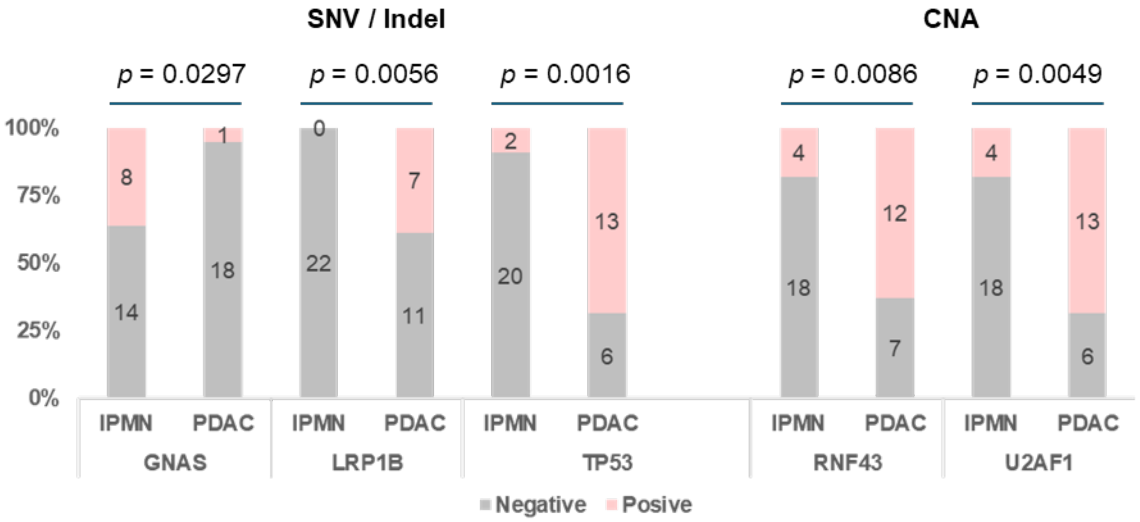

Figure S1C. Specific copy number alterations (CNAs) in IPMN and IPMN-Derived PDACs. Copy number alterations were called using the Battenberg pipeline, and significant regions were identified with GISTIC2. Differences in CNVs between tumour groups were assessed using Fisher's exact test with FDR correction.

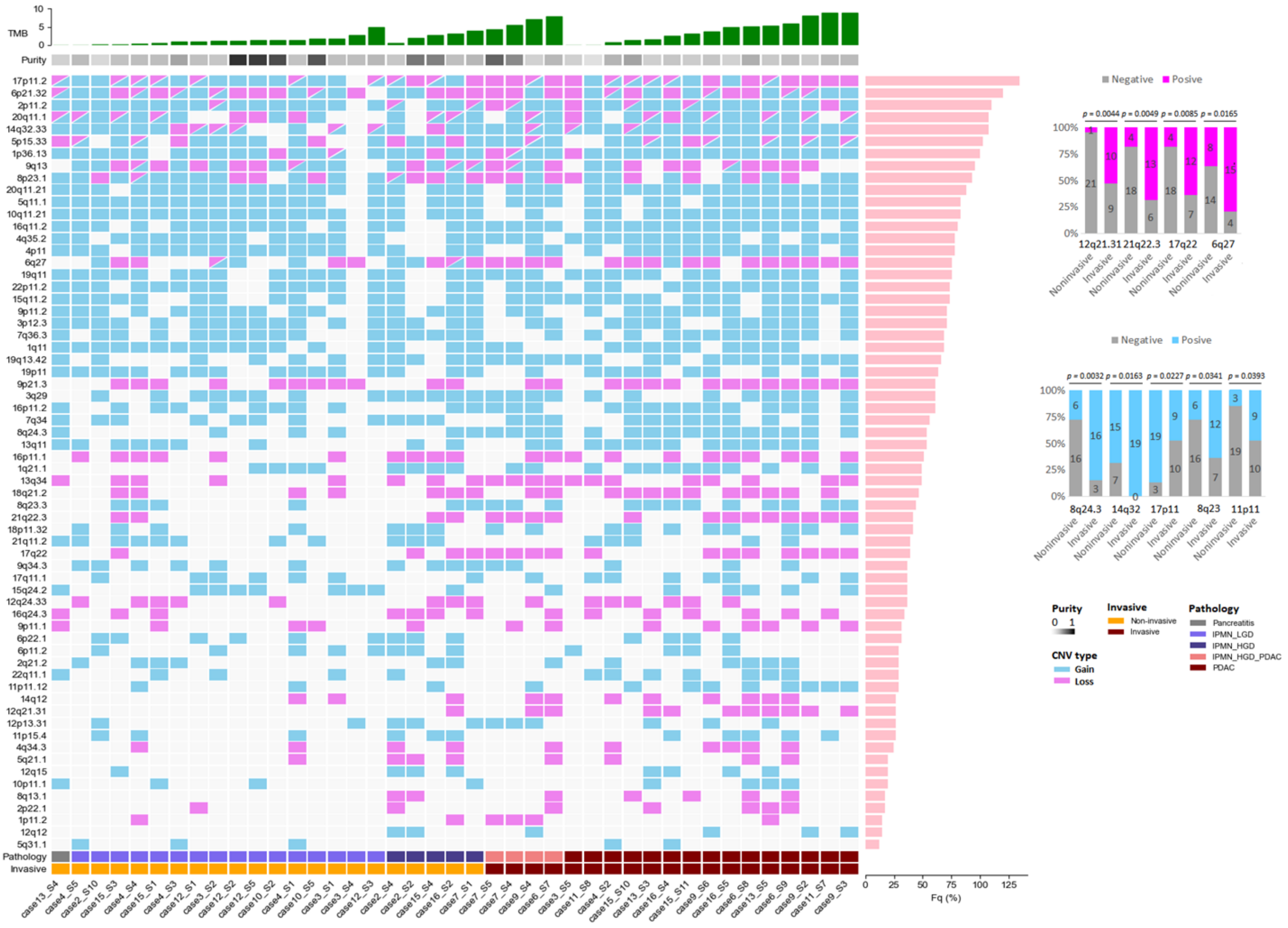

Figure S2. (A) Mutational signature analysis identified 8 single-base substitution (SBS), 7 indel (ID), 6 structural variants (SV), and 4 copy number variation (CNV) COSMIC signatures. The numbers in brackets indicate the number of identified signatures per total number of samples. **(B)** CNV48B is observed across both low- and high-grade lesions and is characterized by marked loss of heterozygosity (LOH). **(C)** We found a moderate and statistically significant positive correlation (*spearman*) between CNV48B activity and the activities of ID1 and ID2 (R = 0.53 and 0.55; p = 0.003 and 0.0002, respectively).

Figure S2A Proportion of Mutational Signature in Each IPMN and IPMN-Derived PDAC. Source data are provided as a Source Data file.

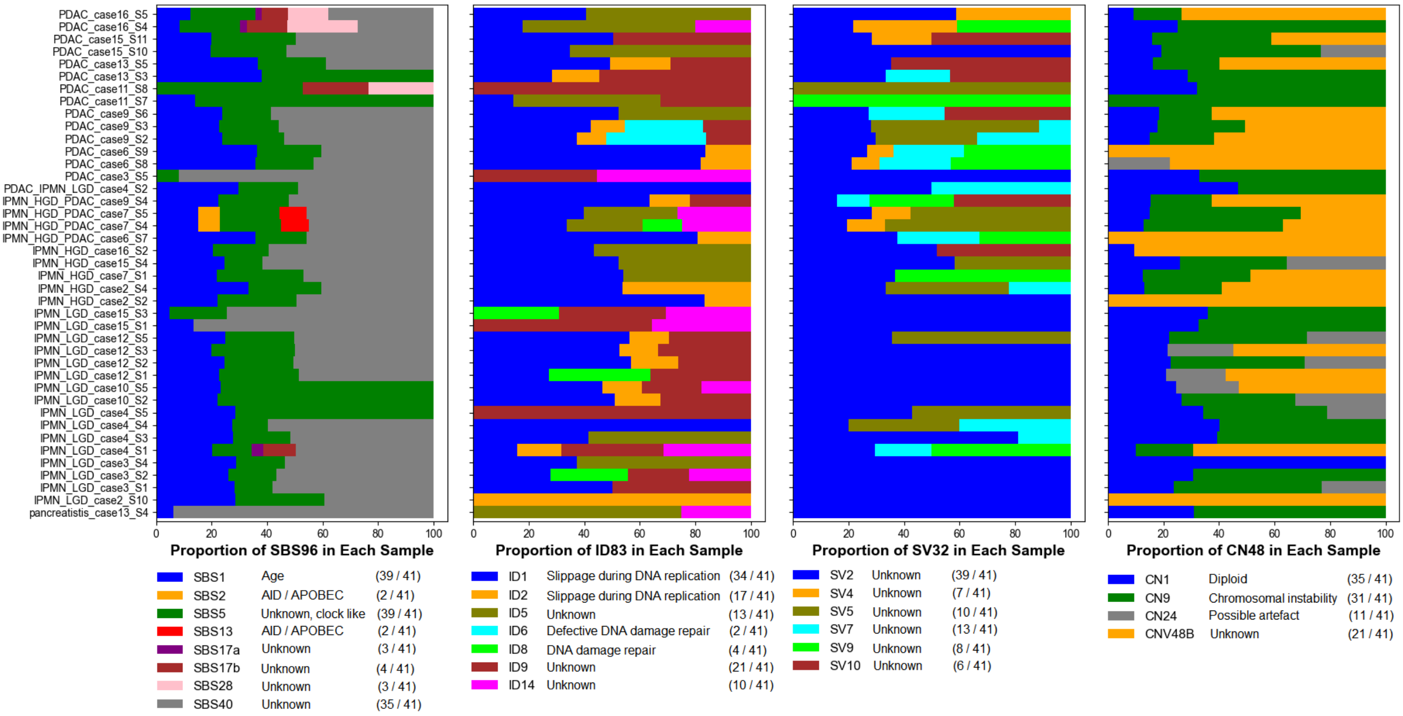

Figure S2B. Profile of the novel CNV signature CN48B

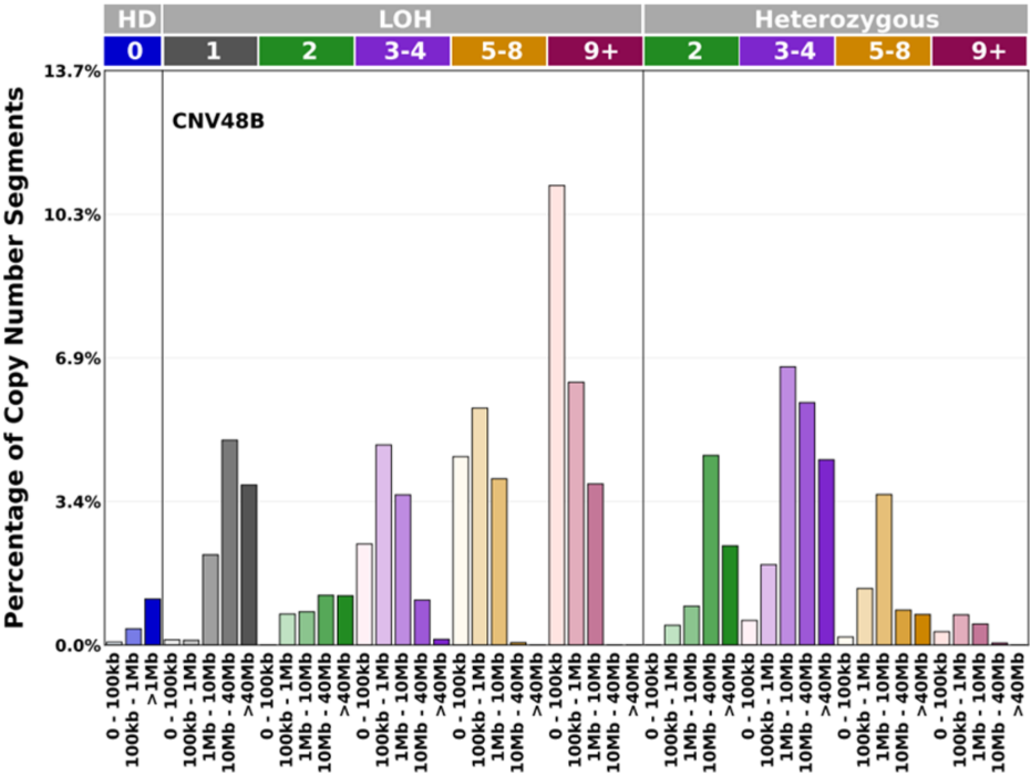

Figure S2C. Correlation analysis of CNV48B vs ID1 and ID2. Source data are provided as a Source Data file.

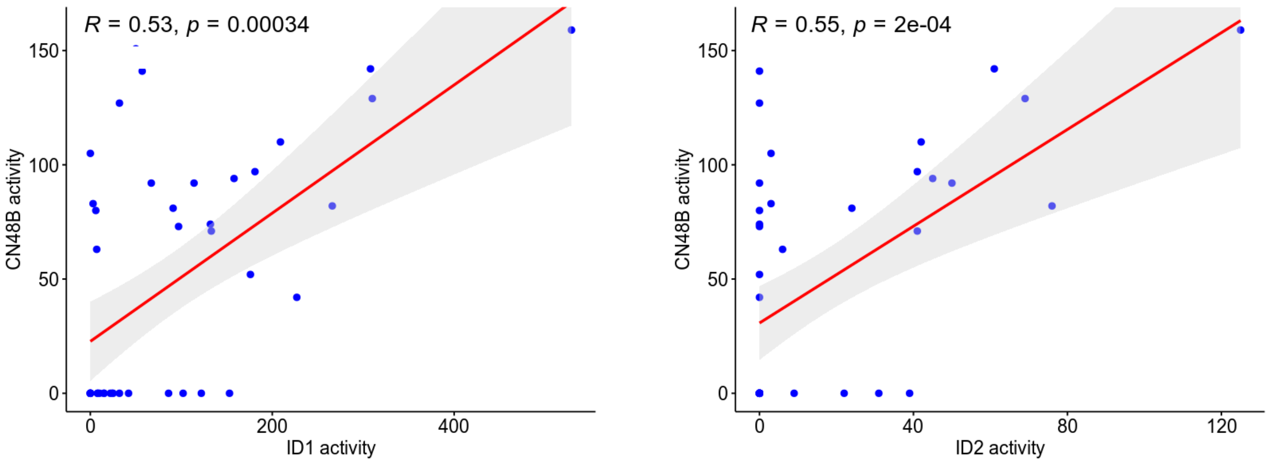

Figure S3. Timing analysis of mutations in PACA from PCAWG (n=229): Using the developed method, SNV mutations were classified into five timing groups, and their numbers and proportions were calculated. A. Raw proportions of timing groups across tumour samples. B. Comparative analysis among timing categories. We performed pairwise comparisons of driver mutation proportions across different timing categories. Most comparisons showed statistically significant differences (adjusted p-values < 0.05), indicating distinct distributions between timing groups. In particular, subclonal mutations were significantly different from early clonal mutations (adjusted p = 0.049). A few comparisons, such as those involving the possiblyLate- cloneEarly and possibly. Late-subclonal group, were not statistically significant, suggesting similarity in mutation proportions for those pairs. C. Analysis of clonal early and subclonal drivers. Focusing on driver mutations with a pooled frequency > 5%, four recurrent drivers, *KRAS*, *TP53*, *CDKN2A*, and *FAT3,* were predominantly observed in the clonal early group. Gene labels in purple, blue and black represent oncogenes, tumour suppressors and ambiguous classifications, respectively. Source data are provided as a Source Data file.

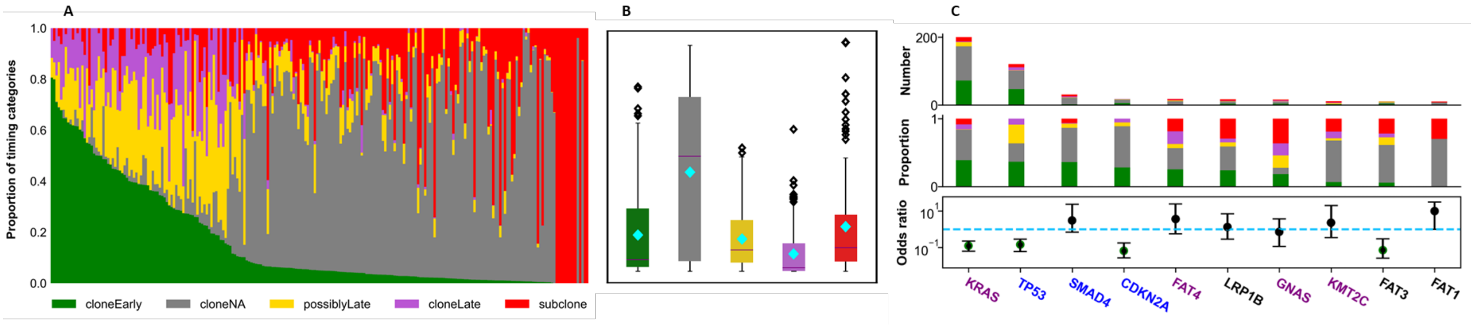

Figure S4: Transcriptome sample correlation heatmaps. The correlation heatmaps show the overall similarity of the expression profiles of samples. We calculated the Euclidean distance between all samples and presented by case.

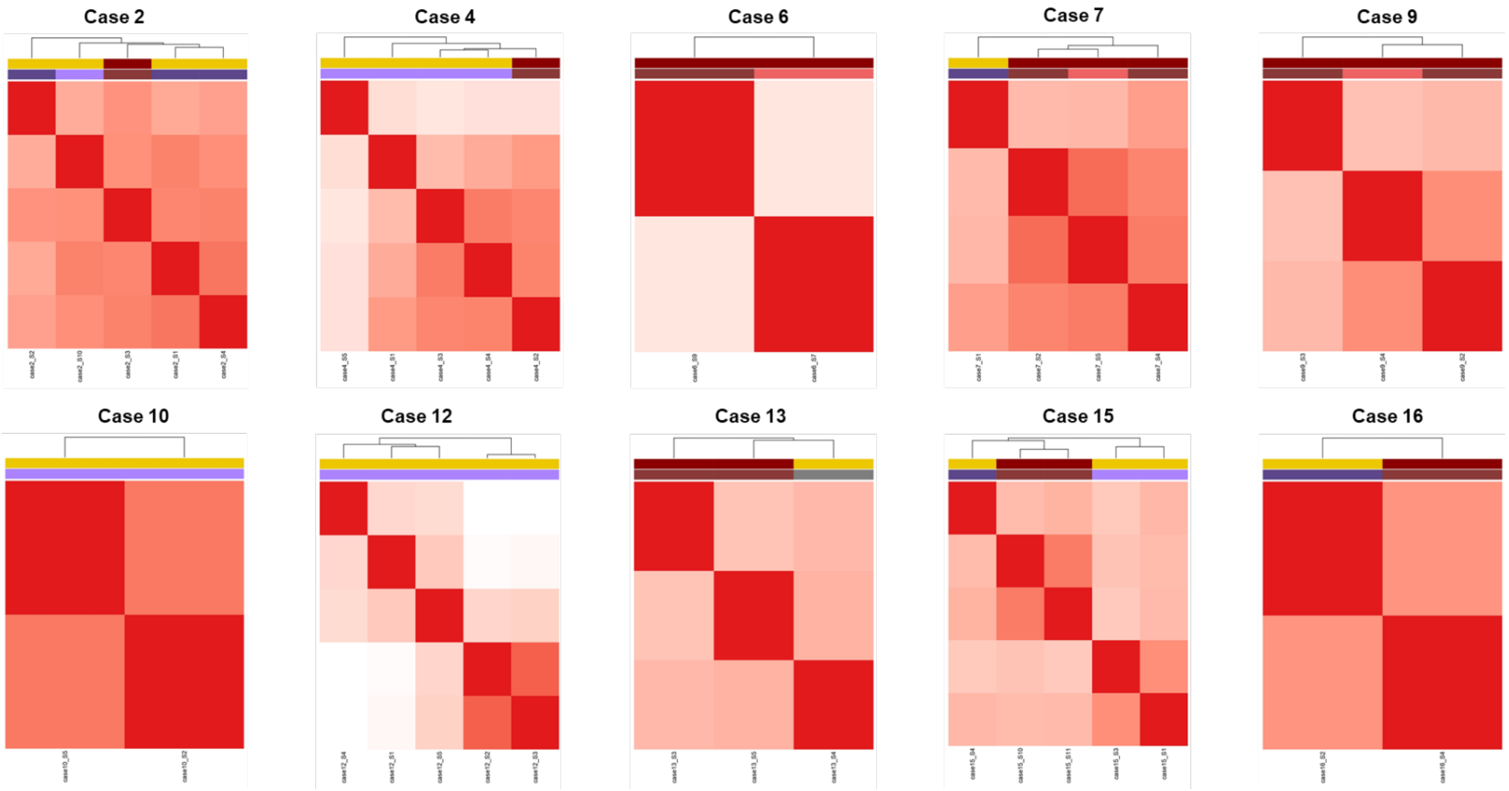

Figure S5: Hallmarks of Cancer. (A) Transcriptional network dynamics comparing non-invasive and invasive samples, showing a selected subset of hallmark pathways typically enriched in pancreatic cancer. (B) Box plots of pathways showing significant differences between non-invasive and invasive samples. Box plots display the median (centre line), interquartile range (box), and minima and maxima (whiskers). Comparisons were based on 35 samples (20 non-invasive, 15 invasive) using a two-sided Wilcoxon rank-sum test ($p < 0.05$). Source data are provided as a Source Data file.

A

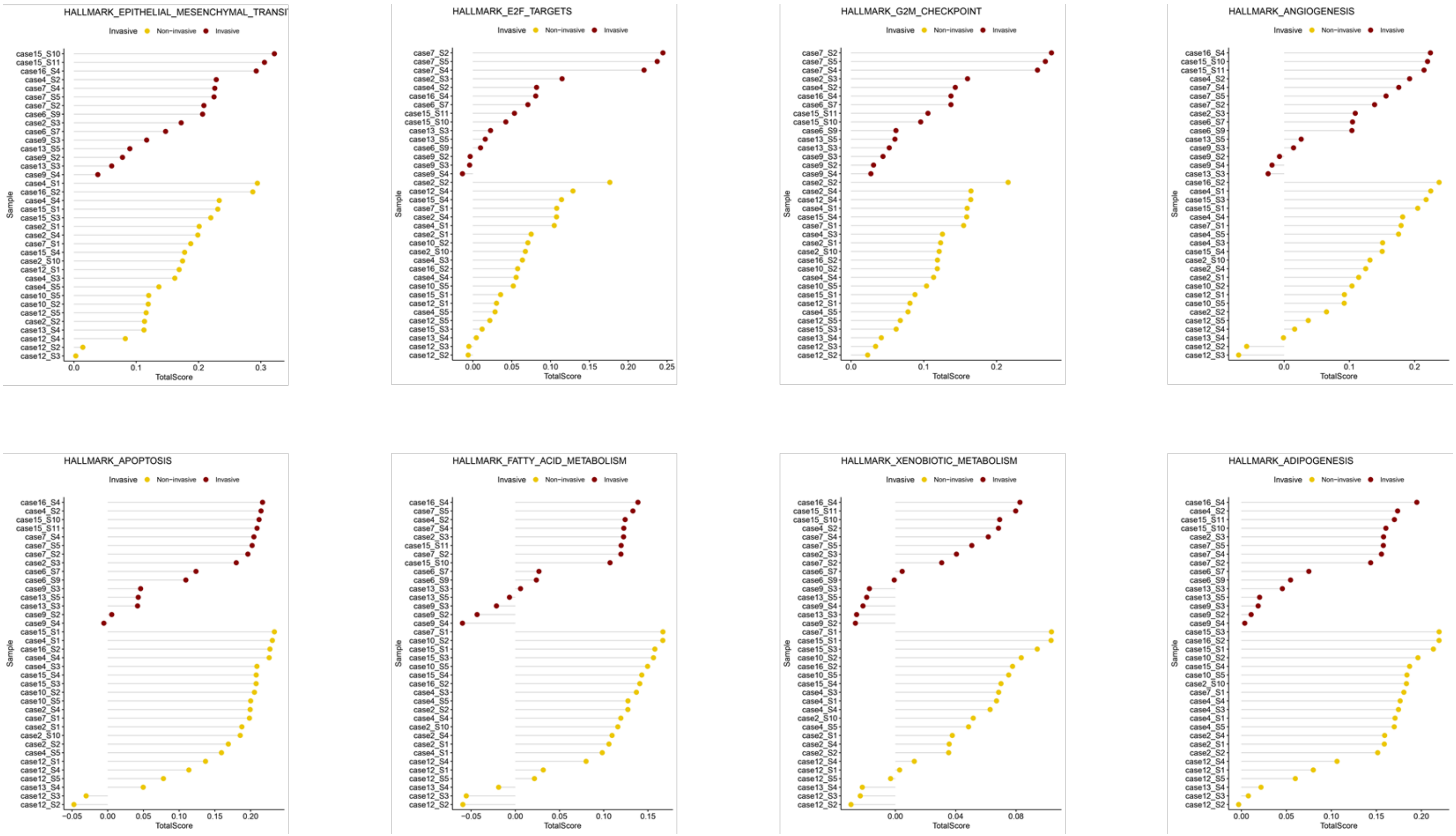

B

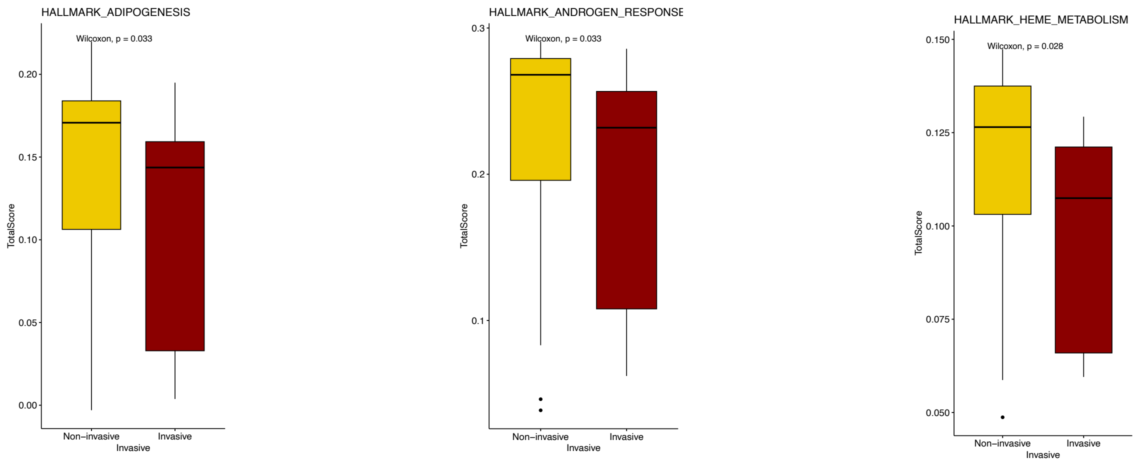

Figure S6: PDAC transcription signatures. Heatmap displaying PDAC transcription signatures for each sample, arranged from classical- (top) to squamous-related signatures (bottom) according to Bailey, Collisson and Moffit. The heatmap also includes gene programs significantly enriched according to Bailey et al. in the classical and squamous subtypes.

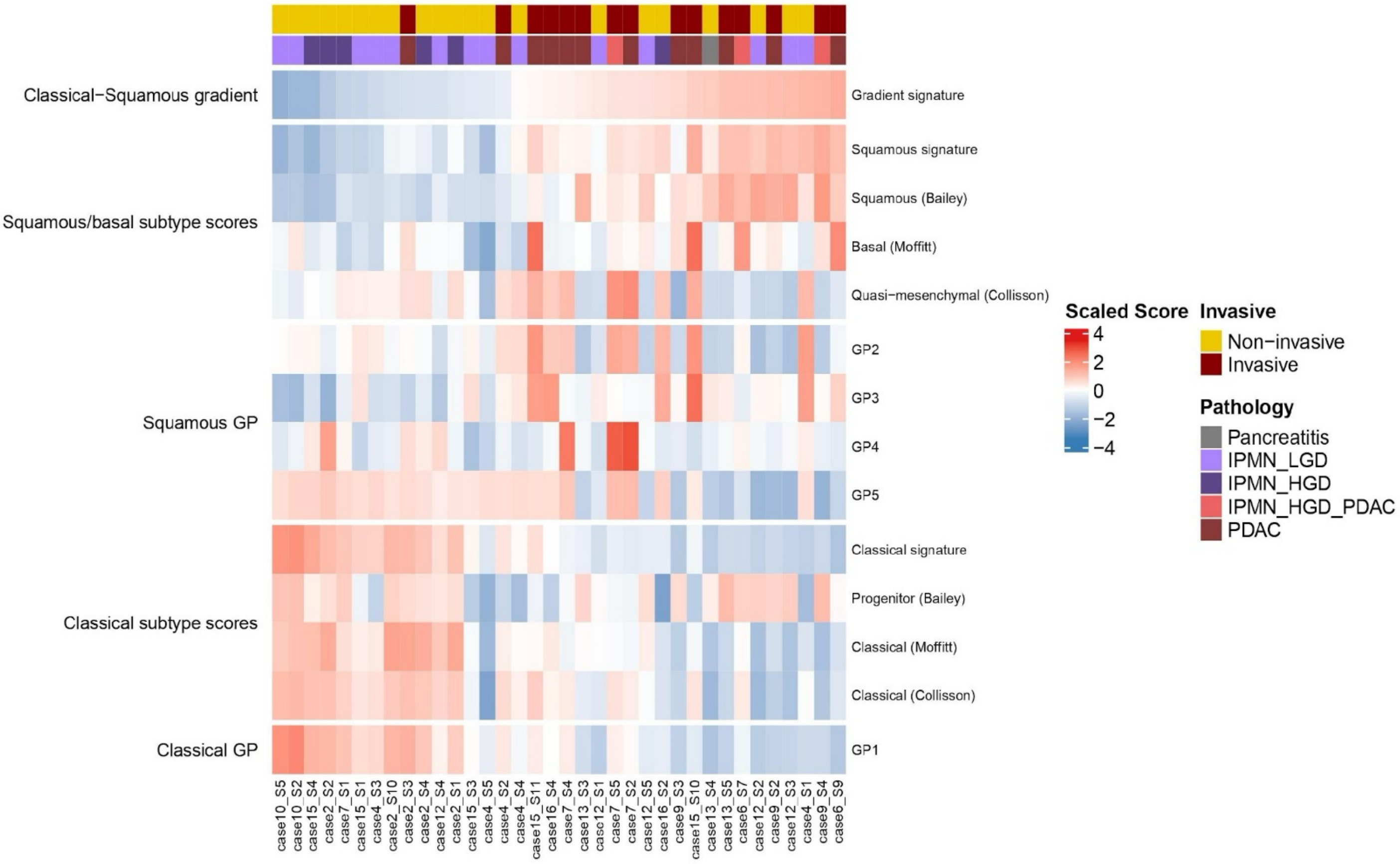

Figure S7

Distribution of ESTIMATE, Stromal, and Immune Scores across samples. Each sample is plotted based on its ESTIMATE score, arranged from the highest to the lowest. The corresponding Stromal and Immune scores for each sample are also depicted**.** Source data are provided as a Source Data file.

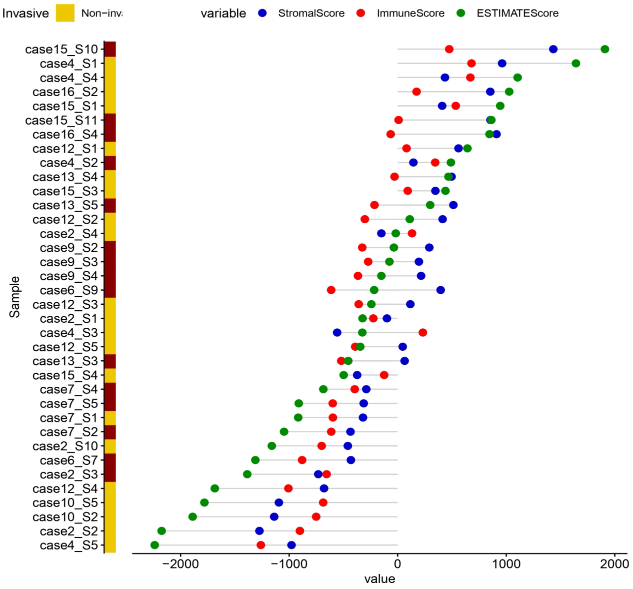

Scatter plots illustrating the relationships between Squamous Score and the ESTIMATE-derived scores. ESTIMATE (Estimation of STromal and Immune cells in MAlignant Tumor tissues using Expression data) is a computational tool designed to predict tumor purity and the presence of infiltrating stromal and immune cells in tumor tissues based on gene expression data. Correlations were assessed using two-sided Spearman's rank correlation tests. B shows a moderate positive correlation between the ESTIMATE Score and the Squamous Score, suggesting that as the overall tumor purity rises, the squamous characteristics might also intensify. C reveals a strong positive correlation between the Stromal Score and the Squamous Score, indicating that samples with a more pronounced stromal component tend to possess heightened squamous features. D displays a weak positive correlation between the Immune Score and the Squamous Score. While this relationship is not statistically significant, it suggests that the degree of immune infiltration in the tumor is not associated with the presence of squamous features.

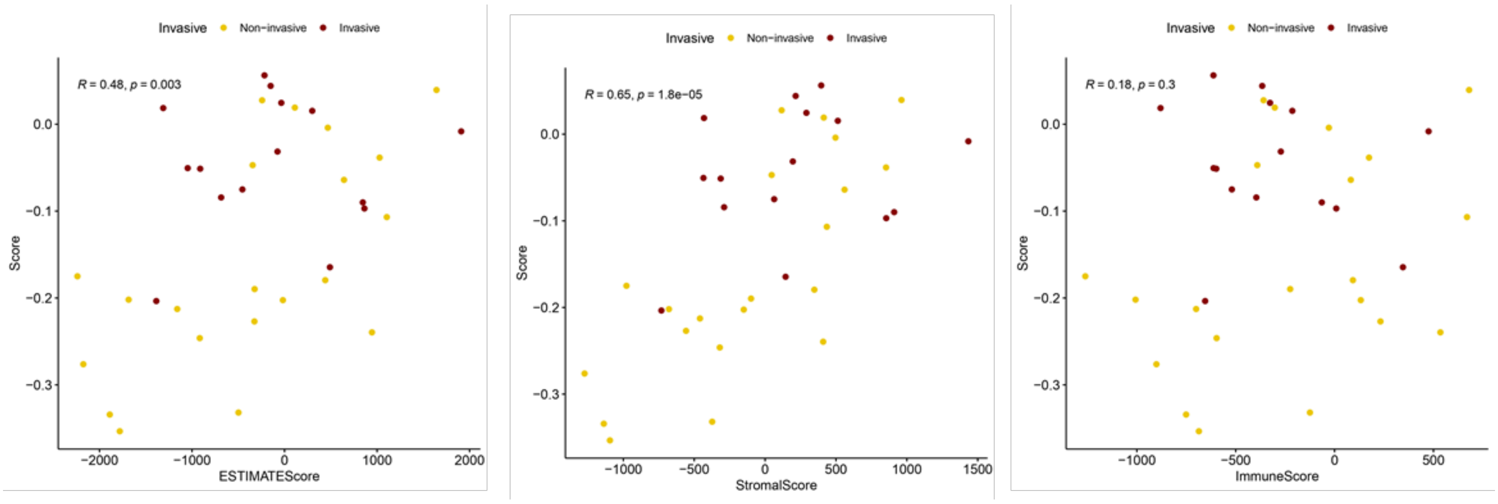

Figure S8: Correlation Between ESTIMATE-derived Scores and Squamous Score in the ICGC Cohort. The scatter plots display the relationships between the ESTIMATE-derived scores (x-axis) and the Squamous Score (y-axis) for 96 ICGC patients subjected to RNAseq. Correlations were assessed using two-sided Spearman's rank correlation tests. **A) Estimate score:** A positive correlation suggests that as the Estimate Score, which reflects overall tumor purity, increases, there's a tendency for the Squamous Score to also rise. **B) Stromal Score**: The stronger positive correlation here implies that samples with a more pronounced stromal component tend to possess heightened squamous features. **C) Immune Score:** The weak correlation suggests that the degree of immune infiltration in the tumor is not a strong determinant of squamous features. The distinct transcriptomic subtypes, as classified by Bailey et al., are highlighted, with the Squamous subtype distinctly segregated. Source data are provided as a Source Data file.

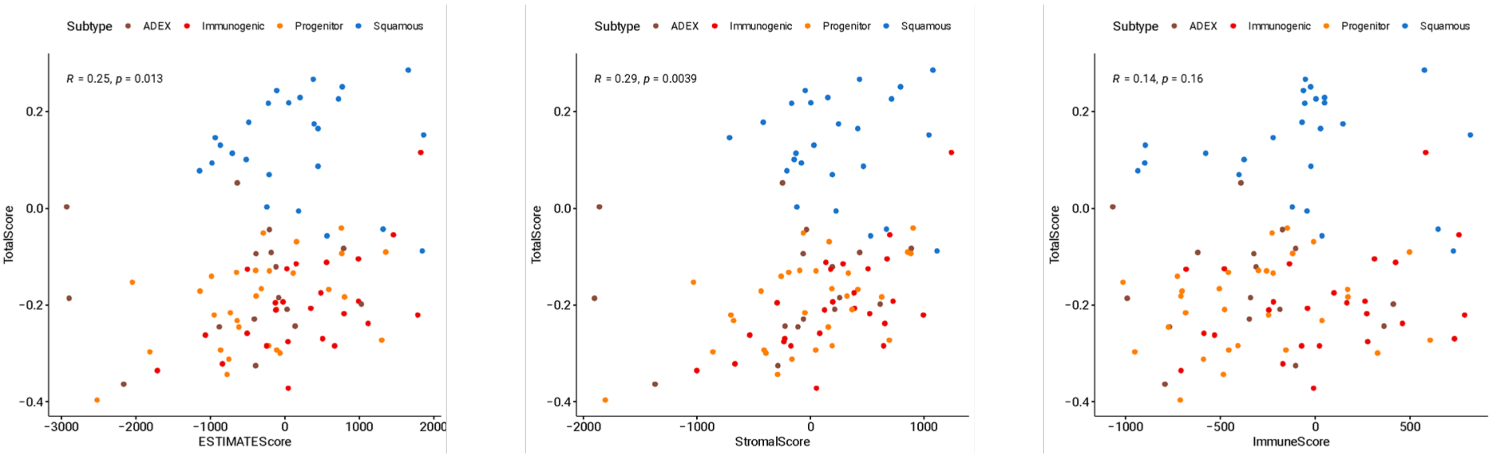

Figure S9. Variations in Immune Cell Composition and ESTIMATE-derived Scores Across Cases. This figure illustrates the dynamic differences in immune cell composition (excluding uncharacterized cells for clarity) and ESTIMATE-derived scores within each individual case. Source data are provided as a Source Data file.

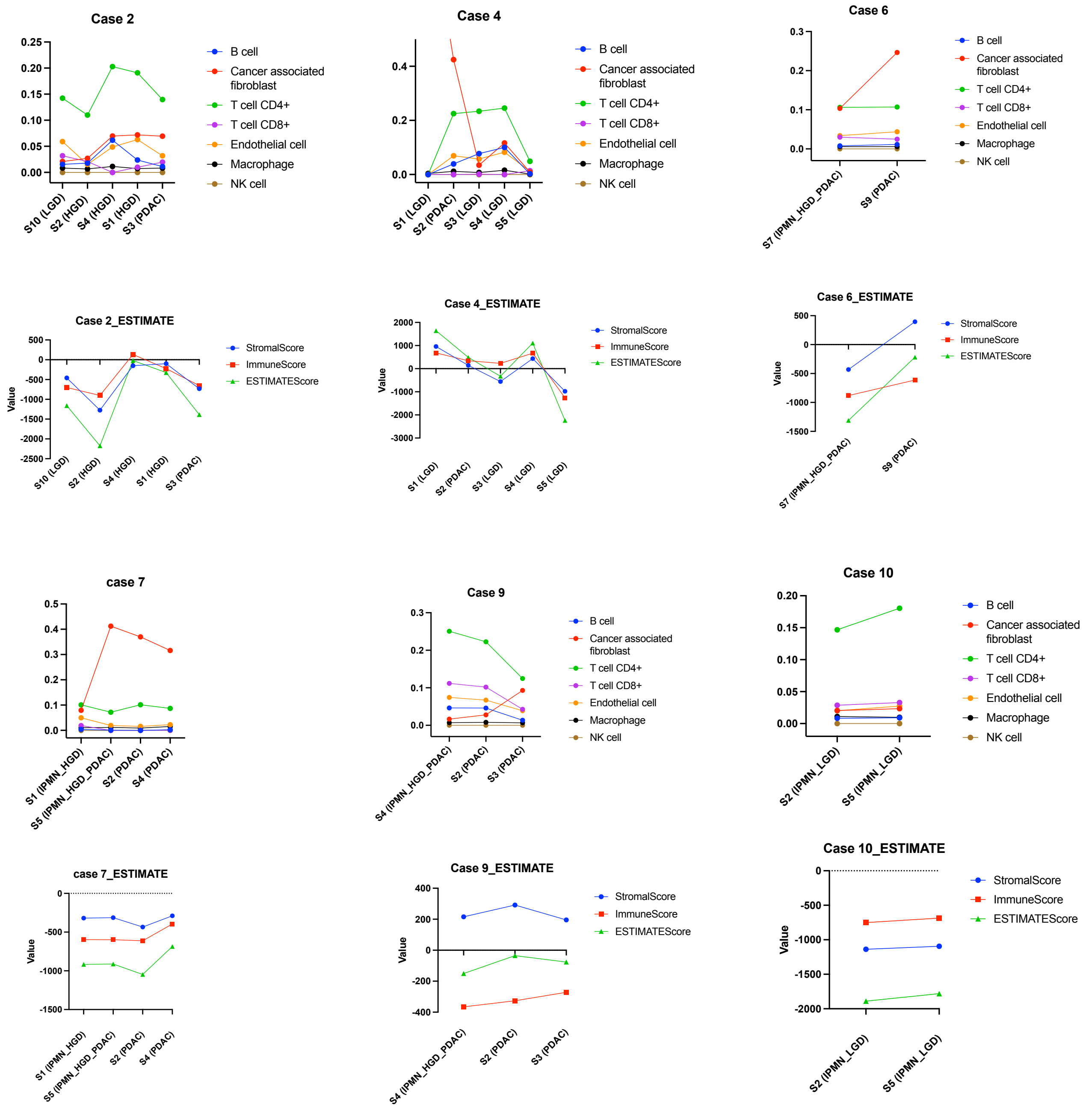

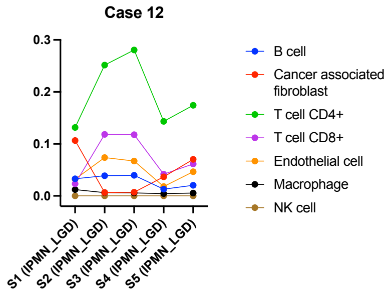
Case 12
0.3
0.2
0.1
0.0
S1 (IPMN_LGD)
S2 (IPMN_LGD)
S3 (IPMN_LGD)
S4 (IPMN_LGD)
S5 (IPMN_LGD)
B cell
Cancer associated fibroblast
T cell CD4+
T cell CD8+
Endothelial cell
Macrophage
NK cell

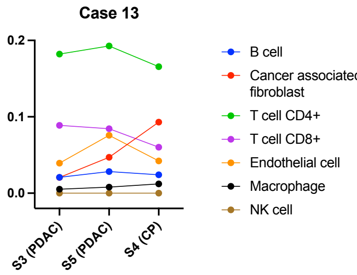
Case 13
0.2
0.1
0.0
S3 (PDAC)
S5 (PDAC)
S4 (CP)
B cell
Cancer associated fibroblast
T cell CD4+
T cell CD8+
Endothelial cell
Macrophage
NK cell

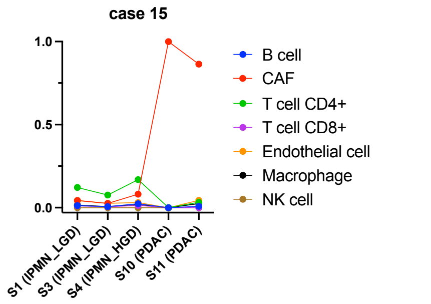
case 15
1.0
0.5
0.0
S1 (IPMN_LGD)
S3 (IPMN_LGD)
S4 (IPMN_HGD)
S10 (PDAC)
S11 (PDAC)
B cell
CAF
T cell CD4+
T cell CD8+
Endothelial cell
Macrophage
NK cell

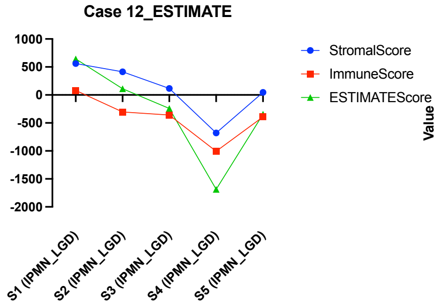
Case 12_ESTIMATE
1000
500
0
-500
-1000
-1500
-2000
S1 (IPMN_LGD)
S2 (IPMN_LGD)
S3 (IPMN_LGD)
S4 (IPMN_LGD)
S5 (IPMN_LGD)
StromalScore
ImmuneScore
ESTIMATEScore

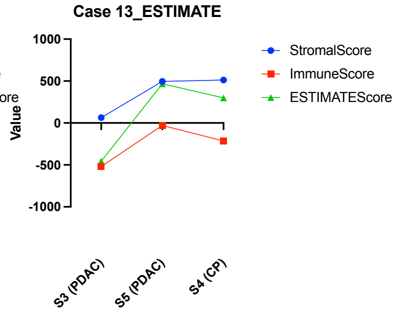
Case 13_ESTIMATE
Value
1000
500
0
-500
-1000
S3 (PDAC)
S5 (PDAC)
S4 (CP)
StromalScore
ImmuneScore
ESTIMATEScore

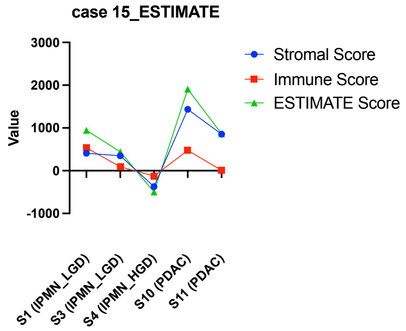
case 15_ESTIMATE
Value
3000
2000
1000
0
-1000
S1 (IPMN_LGD)
S3 (IPMN_LGD)
S4 (IPMN_HGD)
S10 (PDAC)
S11 (PDAC)
Stromal Score
Immune Score
ESTIMATE Score

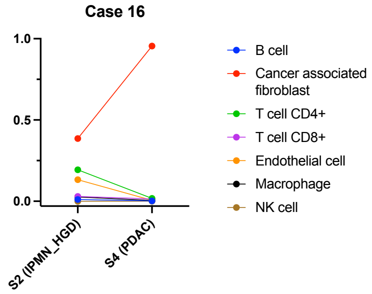
Case 16
1.0
0.5
0.0
S2 (IPMN_HGD)
S4 (PDAC)
B cell
Cancer associated fibroblast
T cell CD4+
T cell CD8+
Endothelial cell
Macrophage
NK cell

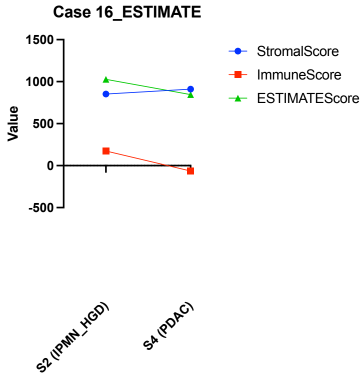
Case 16_ESTIMATE
Value
1500
1000
500
0
-500
S2 (IPMN_HGD)
S4 (PDAC)
StromalScore
ImmuneScore
ESTIMATEScore

Figure S10. (A) Comparison of tumour microenvironment (TME) cell proportions between tumours evolving from a single most recent common ancestor (MRCA) and those with multiple independent clones (MIC). **(B)** Comparison of ESTIMATE Stromal Score, Immune Score, and overall ESTIMATE Score between MRCA and MIC cases. Statistical analysis using a two-sample t-test showed that the levels of cell types B, Macrophage and CD4^{+} T did not differ significantly between tumours following pathway 1 and pathway 2. However, the proportion of CD8^{+} T cell was significantly higher in tumours that progressed via pathway 1. Source data are provided as a Source Data file.

A

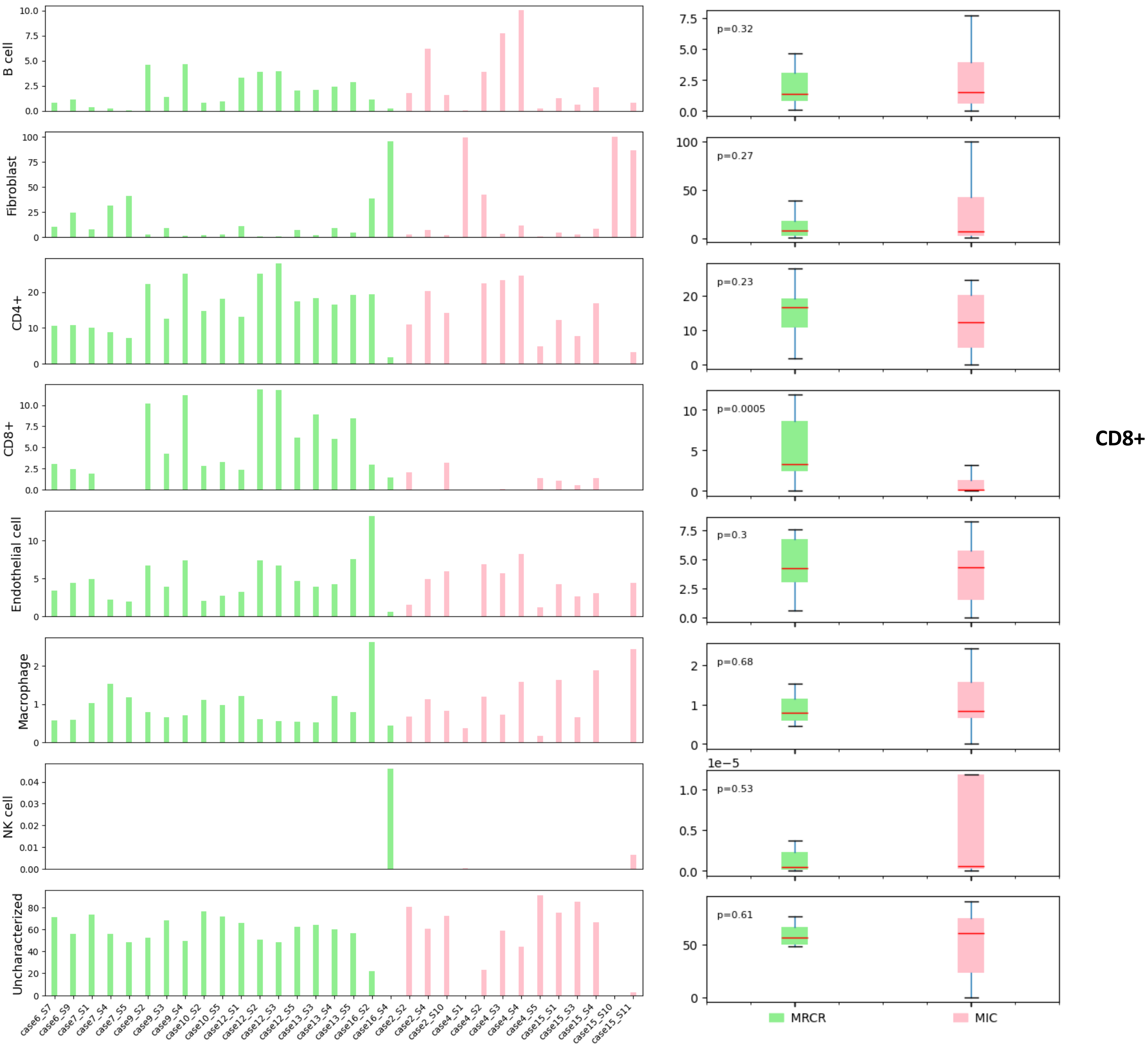

B

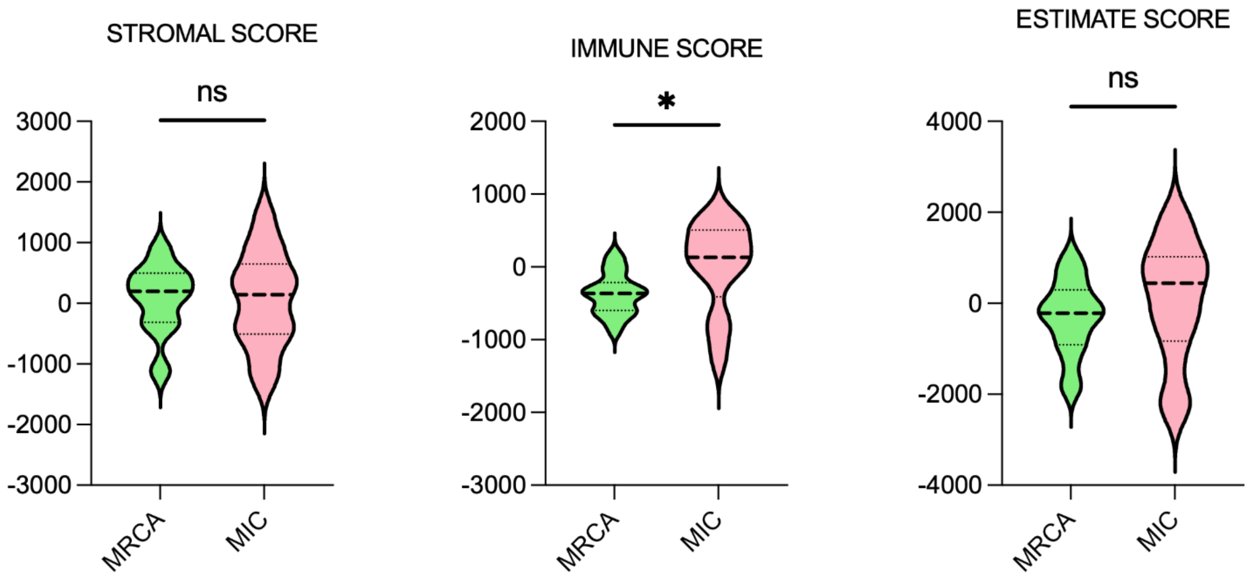
STROMAL SCORE
ns
3000
2000
1000
0
-1000
-2000
-3000
MRCA
MIC
IMMUNE SCORE
*
2000
1000
0
-1000
-2000
-3000
MRCA
MIC
ESTIMATE SCORE
ns
4000
2000
0
-2000
-4000
MRCA
MIC

Supplementary Methods

Multiregional sampling protocol

All tissue samples were immediately snap-frozen in liquid nitrogen post-collection and stored at -80°C. DNA and RNA were extracted from bulk frozen tissues using an AllPrep DNA/RNA Kit (Qiagen), and from blood samples using a Nucleon Genomic Extraction kit (Gen-Probe), following the manufacturer's instructions. DNA and RNA quantification and quality control were conducted using both Nanodrop and High Sensitivity Qubit (Thermo Fisher Scientific).

Hematoxylin and eosin-stained frozen sections with histologically confirmed IPMN or PDAC samples, as determined by an expert pathologist, and with an adequate amount of DNA and RNA, were deemed suitable for sequencing. All H/E slides were digitised for a second review by an expert pathologist and for cellular quantification.

Specific immunohistochemical staining on FFPE diagnostic slides of non-invasive IPMNs and the corresponding PDAC was obtained following standardized procedures[1-3], and evaluated as per manufacturers' instructions. For all cases, the following antibodies were tested: P53 (clone: DO-7; 1:50 dilution; Novocastra/UK), SMAD4 (B-8; 1:1000; Santa Cruz/USA) and, for mismatch repair (MMR) proteins, MLH1 (clone: ES05; dilution 1:30; Dako / Germany), PMS2 (MRQ-28, 1:150, Cell Marque Impath Menarini / Italy), MSH2 (FE11; 1:30, Dako), and MSH6 (EP49; 1:100, Dako).

Read alignment and somatic variant calling

Lanes of paired raw FASTQ reads from each sample were merged through fastqtobam tool and then aligned to human reference genome (hg38) using Sanger bwamem2 mapping workflow cgpWGS330 (ftp://ftp.sanger.ac.uk/pub/cancer/dockstore/human/GRCh38_hla_decoy_ebv/core_ref_GRCh38_hla_decoy_ebv.tar.gz).

Somatic SNVs and InDels were identified from matched normal and tumour pairs using Sanger cgpwgs210 within a singularity (https://github.com/cancerit/dockstore-cgpwgs/wiki/ Running-under-singularity) and Mutect2 from GATK 4.1.8 (https://gatk.broadinstitute.org/hc/en-us/sections/ 360009656231-4-1-8-0). Driver genes of interest were identified as missense and nonsense SNVs, and as frameshift deletions / insertions and essential splice site deletions.

In order to generate VCF datasets for further analysis, mutational variants from these tools were selected with pass parameter and finally intersected using bcftools 1.11. In parallel, Breakpoint variants were isolated and assembled by cgpwgs210-BARSS. The SV files across all samples were filtered via SURVIVOR software and processed per sample using Python package Scikit-Allel to compute aggregated counts of each SV type:

deletion, inversion, translocation, tandem duplication and multiple features. Fifty known drivers were queried from the final relevant datasets based on COSMIC cancer gene census[4,5].

Mutation and copy number signature profiling

Mutation signatures were analysed by using SigProfilerExtractor based on a non-negative matrix factorization (NNMF) framework (v0.0.5.77; https://github.com/AlexandrovLab/ SigProfilerExtractor). Signatures from De novo extraction and decomposition were profiled as single-base substitution (SBS96), double-base substitution (DBS78) and small insertion-deletion (ID83)[6]. Using ASCAT matrix generated from CNA datasets, copy number signatures were extracted by SigProfilerExtractor[7]. Based on the updated known signatures as in COSMIC database, SigProflerAssignment was utilised to retrieve decomposed signatures.

In order to minimise NNMF artefact potentials, initial signature extraction was performed with all available samples simultaneously[8] and each single signature assignment per sample was finally determined by the probability matrix.

Mutation clustering and phylogenetic tree reconstruction

To model subclonal structure and construct phylogenetic tree from multiple normal-tumour matched samples, we combined various genomic datasets and applied a number of bioinformatics tools. For each sample, mutation allele fractions (MAF) of SNV and Indel were prepared by using alleleCounter and vafCorrect. Together with copy number and cellularity, these outcomes were submitted for clustering mutations to their mutation copy number using a previously described Bayesian Dirichlet process (DPClustering)[9], (v2.2.8; https://github.com/Wedge-lab/dpclust/releases). This estimation per sample was extended into n dimensions for 12 IPMN-PDAC cohorts with n related samples, where the numbers of mutant reads obtained from multiple related samples were modelled as independent binomial distributions. Variants from each cohort with an upper CCF boundary above and below 1 were considered to be clonal and subclonal respectively[10].

To determine the most likely phylogenetic tree we applied the "pigeon-hole" principle (PHP) or 'sum' rule and 'crossing rule' to mutational clusters within individual samples[10-12]. The sum rule asserts that if a subclone A is ancestral to both subclones B and C and if the summed CCFs of B and C exceed the CCF of A in any sample, the relationship between the subclones must be linear; whereas the crossing rule asserts that if the CCF of B is higher than the CCF of C in sample X and the CCF of B is lower than the CCF of C in sample Y, then B and C are constructed as separate branches of the phylogenetic tree[13].

Annotation of the trees with mutations and signatures

To annotate each tree with oncogenic or putative oncogenic alterations, in-house programs were utilised to connect multiple genomic variants and identify specific significance, including SNV, Indels, SV, CNA, mutation signature and cluster assignment information from mutation callers, Battenberg, SigProfilerExtractor and multisample DPClust.

SNV and Indel Signatures were assigned to each branch in the phylogenetic tree using the following steps:

1. Mutation identification: we obtained the genomic coordinates of all somatic mutations from whole-genome sequencing (WGS) data.

2. Variant clustering: Using multidimDPClust, we performed multidimensional clustering of variants, incorporating confidence intervals for assignment. This process generated mutational clusters along with their corresponding genomic coordinates. We included clusters containing at least 1% of the total mutations.

3. Phylogenetic reconstruction: we reconstructed the phylogenetic tree based on the outputs from multidimDPClust, assigning mutations (along with their genomic coordinates) to specific branches.

4. Signature extraction: we identified single-nucleotide variant (SNV) signatures (SBS96) and insertion-deletion (Indel) signatures (ID83) using COSMIC methodologies. The genomic coordinates of mutations contributing to each mutational signature were extracted.

5. Assignment of signatures to tree branches: The probability of each mutational signature corresponding to a clonal cluster (tree branch) was derived by merging mutation coordinates from steps 3 and 4 for each multi-sample case. For each cluster, we used the probability of each mutation context being assigned to a signature and summed these probabilities across all mutations within the cluster.

(21-Assignment of Signatures to Clustered Branches:
https://gitfront.io/r/xtgitfhe2/8f2rTdyZs1S9/IPMNPDACpaperArchive/

Annotation of the trees with SVs and CNVs

SV clusters were identified by matching their genomic coordinates with those of SNV/indel clusters from the same sample. SV drivers were then assigned to the corresponding branches. Similarly, CNV clusters were assigned to tree branches by genomic coordinate matching to SNV/indel clusters.

(24-Assignment of SV (gene-driver) to Clustered Branches and 25-Assignment of CNV (gene-driver) to Clustered Branches,

https://gitfront.io/r/xtgitfhe2/8f2rTdyZs1S9/IPMNPDACpaperArchive/)

Battenberg-DPClust Timing of SNV and Indel Mutations (BBD timing)

To infer the likely timing and clonal status of mutations within the tumour, we categorized them into six groups (from clonal early to subclonal) using the BBD timing approach, which integrates cancer cell fraction (CCF) estimates and local copy number information derived from Battenberg and DPClust (https://github.com/Wedge-lab). Mutations with a CCF ≤ 0 were designated as unspecialized (unSp), indicating likely sequencing artefacts or biologically insignificant variants with minimal representation in the cancer cell population. Mutations with a CCF between 0 and 0.95 were classified as subclonal, representing variants likely emerging after the initial clonal expansion.

Mutations with a CCF of at least 0.95, indicative of high cancer cell prevalence, were further subdivided based on copy number features and chromosomal distribution. If either of the major alleles (nMaj1 or nMaj2) had a copy number greater than 1 and the mutation was present on more than one chromosome copy (noChrsBearingMut > 1), the mutation was classified as clonal early (cloneEarly), suggesting early clonal mutations that underwent chromosomal duplication. If all available alleles were greater than 1 (nMaj1, nMaj2, nMin1 and nMin2 all >1 or nMaj1 and nMaj2 > 1, nMin1 and nMin2=0), and the mutation was detected on just one chromosome copy (noChrsBearingMut ≤ 1), the mutation was classified as clonal late. If a mutation was observed on just one chromosome copy (noChrsBearingMut ≤ 1) but it was uncertain whether that copy had undergone a copy number gain (nMaj1 or nMaj2 > 1, nMin1 and nMin2 = 1), the mutation was classified as possibly clonal late (possiblylate). Any remaining mutations, which did not clearly fall into the above categories, were grouped as 'clonalNA' . https://gitfront.io/r/xtgitfhe2/8f2rTdyZs1S9/IPMNPDACpaperArchive/

RNAseq analysis

Sequencing quality of fastq files was assessed with FastQC (https:// github.com/s-andrews/FastQC) (v 0.11.9) and files were processed with fastp[14] (v 0.21.0) using default settings. Quantification was performed against GRCh38 using Salmon[15] (v 1.4.0). Salmon quantification results were imported into a DESeqDataSet using DESeq2[16] (v 1.38.3). Transcripts were mapped to genes using EnsDb.Hsapiens.v86[17] (v 2.99.0). Read count data were filtered to retain only those with normalised counts >= 5 in at least 18 samples (18,409 genes

retained). Reads were transformed using the DESeq2 'vst' function. PCA was performed with plotPCA (DESeq2). Potential confounders such as histological grade, epithelial subtype, and sequencing batch were evaluated during downstream analyses, and batch correction was applied during normalization where appropriate.

Scores were calculated for 50 Hallmark[18] gene sets from MSigDB. Scores were calculated using the singscore[19,20] package and the VST transformed counts. A transcriptomic gradient score was derived for each sample using single-sample gene set enrichment analysis (ssGSEA) based on previously published squamous and classical gene signatures (Bailey et al., Nature 2016; Brunton et al., Cell Reports 2020). The final score was computed as the difference between squamous and classical enrichment values, providing a continuous metric of transcriptional subtype.

Cell type proportions were estimated using the EPIC method as implemented in the immunedeconv[21] package using TPM counts. The ESTIMATE[22] method was used to calculate stromal, immune and overall ESTIMATE scores, implemented in the estimate (https://bioinformatics.mdanderson.org/ estimate/rpackage.html) package.

Data Processing and Statistical Analysis

Pipeline running, data processing, statistical analysis and visualisation were mainly performed in R and Python programming languages based on High Performance Computing (HPC) cluster, The University of Manchester.

To determine an association between lesion grade and the presence of mutational types, we applied Fisher's Exact Test for genomic alterations and the Mann–Whitney Test for expression and deconvolution data across different tumor stages. To assess differences in cell count data between Clusters 1 and 2, median values from samples of each case were considered, while for differential gene expression between transcriptomic clusters, two-sided Student's *t*-tests were used. Additionally, to compare the overall number of SNVs, indels, SVs, and TMB between IPMN and PDAC, we used the Fisher–Pitman Permutation Test. A p value of less than 0.05 was considered statistically significant.

Multiple hypothesis testing was controlled using the Benjamini–Hochberg procedure, with FDR < 0.05 considered statistically significant. Specifically, we applied Fisher–Pitman permutation tests to compare the number of SNVs, indels, and structural variants (SVs) across tumor types and genomic regions; Fisher's exact tests to compare the frequency of driver mutations (including SNVs, indels, SVs, and CNAs) and SV signatures across tumor types; and permutation-based Wilcoxon tests for pairwise comparisons of timing group proportions.

To determine whether specific cancer driver mutations are preferentially clonal early or subclonal, we applied a bootstrap resampling approach to estimate odds ratios (ORs) between subclonal and early clonal occurrences across tumors. For each driver, we computed the median OR and the 95% confidence interval (CI), defined as the 2.5th to 97.5th percentiles of the bootstrap distribution. A driver was considered significantly enriched in subclonal or clonal early mutations if the 95% CI of the OR did not include 1. Specifically, a CI greater than 1 indicated significant enrichment in subclonal timing, a CI less than 1 indicated significant enrichment in early clonal timing, and a CI that overlapped 1 indicated no significant enrichment.

Data availability

Whole-genome sequencing (WGS) and RNA-seq raw data have been deposited in the European Genome-phenome Archive (EGA) / under accession number EGAS50000001182 (https://submission.ega-archive.org/studies/EGAS50000001182)

Data Analysis and Code availability

The Code and data used for the analysis are available at:

https://gitfront.io/r/xtgitfhe2/8f2rTdyZs1S9/IPMNPDACpaperArchive/ [gitfront.io].

1. Luchini, C. *et al.* CD71 in Gestational Pathology: A Versatile Immunohistochemical Marker With New Possible Applications. *Appl Immunohistochem Mol Morphol* **24**, 215-20 (2016).
2. Mafficini, A. *et al.* Juvenile polyposis diagnosed with an integrated histological, immunohistochemical and molecular approach identifying new SMAD4 pathogenic variants. *Fam Cancer* **21**, 441-451 (2022).
3. Mafficini, A. *et al.* Integrative characterization of intraductal tubulopapillary neoplasm (ITPN) of the pancreas and associated invasive adenocarcinoma. *Mod Pathol* **35**, 1929-1943 (2022).
4. Sondka, Z., Bamford, S., Cole, C.G., Ward, S.A., Dunham, I. & Forbes, S.A. The COSMIC Cancer Gene Census: describing genetic dysfunction across all human cancers. *Nat Rev Cancer* **18**, 696-705 (2018).
5. Martinez-Jimenez, F. *et al.* A compendium of mutational cancer driver genes. *Nat Rev Cancer* **20**, 555-572 (2020).
6. Alexandrov, L.B. *et al.* The repertoire of mutational signatures in human cancer. *Nature* **578**, 94-101 (2020).
7. Steele, C.D. *et al.* Signatures of copy number alterations in human cancer. *Nature* **606**, 984-991 (2022).

8. Ansari-Pour, N. *et al.* Whole-genome analysis of Nigerian patients with breast cancer reveals ethnic-driven somatic evolution and distinct genomic subtypes. *Nat Commun* **12**, 6946 (2021).
9. Dentro, S.C., Wedge, D.C. & Van Loo, P. Principles of Reconstructing the Subclonal Architecture of Cancers. *Cold Spring Harb Perspect Med* **7**(2017).
10. Gundem, G. *et al.* The evolutionary history of lethal metastatic prostate cancer. *Nature* **520**, 353-357 (2015).
11. Nik-Zainal, S. *et al.* The life history of 21 breast cancers. *Cell* **149**, 994-1007 (2012).
12. Bolli, N. *et al.* Heterogeneity of genomic evolution and mutational profiles in multiple myeloma. *Nat Commun* **5**, 2997 (2014).
13. Noorani, A. *et al.* Genomic evidence supports a clonal diaspora model for metastases of esophageal adenocarcinoma. *Nat Genet* **52**, 74-83 (2020).
14. Chen, S., Zhou, Y., Chen, Y. & Gu, J. fastp: an ultra-fast all-in-one FASTQ preprocessor. *Bioinformatics* **34**, i884-i890 (2018).
15. Patro, R., Duggal, G., Love, M.I., Irizarry, R.A. & Kingsford, C. Salmon provides fast and bias-aware quantification of transcript expression. *Nat Methods* **14**, 417-419 (2017).
16. Love, M.I., Huber, W. & Anders, S. Moderated estimation of fold change and dispersion for RNA-seq data with DESeq2. *Genome Biol* **15**, 550 (2014).
17. Rainer, J., Gatto, L. & Weichenberger, C.X. ensembldb: an R package to create and use Ensembl-based annotation resources. *Bioinformatics* **35**, 3151-3153 (2019).
18. Liberzon, A., Birger, C., Thorvaldsdottir, H., Ghandi, M., Mesirov, J.P. & Tamayo, P. The Molecular Signatures Database (MSigDB) hallmark gene set collection. *Cell Syst* **1**, 417-425 (2015).
19. Foroutan, M., Bhuva, D.D., Lyu, R., Horan, K., Cursons, J. & Davis, M.J. Single sample scoring of molecular phenotypes. *BMC Bioinformatics* **19**, 404 (2018).
20. Bhuva, D.D., Cursons, J. & Davis, M.J. Stable gene expression for normalisation and single-sample scoring. *Nucleic Acids Res* **48**, e113 (2020).
21. Sturm, G., Finotello, F. & List, M. Immunedeconv: An R Package for Unified Access to Computational Methods for Estimating Immune Cell Fractions from Bulk RNA-Sequencing Data. *Methods Mol Biol* **2120**, 223-232 (2020).
22. Yoshihara, K. *et al.* Inferring tumour purity and stromal and immune cell admixture from expression data. *Nat Commun* **4**, 2612 (2013).
