# Supplementary material for: Clonal evolutionary analysis reveals patterns of malignant transformation of Intraductal Papillary Mucinous Neoplasms of the pancreas
Source: Nat Commun. 2026 Mar 4;17:3427. doi: 10.1038/s41467-026-69762-w (PMC13076896; doi:10.1038/s41467-026-69762-w)
Supplement: Supplementary file 2 — Description of Additional Supplementary Files [file 41467_2026_69762_MOESM2_ESM.pdf]

Description of Additional Supplementary Files

Supplementary Data 1: Sample manifest. List of all IPMN and PDAC samples included in the study, including patient ID, specimen type (LGD,HGD, PDAC), and sequencing metadata.

Supplementary Data 2: SNV, Indels, Cellularity, and Tumor Mutational Burden (TMB). Summary of single nucleotide variants (SNVs), small insertions/deletions (indels), estimated tumor cellularity, and tumor mutational burden per sample.

Supplementary Data 3: Structural Variant (SV) counts Number and type of structural variants identified across all samples.

Supplementary Data 4: KRAS codon amino acid changes KRAS mutation details, including codon position, nucleotide change, and resulting amino acid substitution for each sequenced sample.

Supplementary Data 5: Copy Number Variation (CNV) Fisher Test. Results of Fisher's exact test comparing copy number variation frequencies between histological subgroups, with associated p-values and adjusted significance levels.

Supplementary Data 6: CCF clusters of SNV and indel mutations identified from multiple samples using DPC analysis. Each row represents a mutation cluster, and the numbers in the columns correspond to the CCF values of that cluster in each sample.
