# Supplementary material for: Clonal evolutionary analysis reveals patterns of malignant transformation of Intraductal Papillary Mucinous Neoplasms of the pancreas
Source: Nat Commun. 2026 Mar 4;17:3427. doi: 10.1038/s41467-026-69762-w (PMC13076896; doi:10.1038/s41467-026-69762-w)
Supplement: Supplementary file 9 — Reporting summary [file 41467_2026_69762_MOESM9_ESM.pdf]

Corresponding author(s): Antonio Pea; David Chang; David Wedge
Last updated by author(s): Nov 3 2025

Reporting Summary

Nature Portfolio wishes to improve the reproducibility of the work that we publish. This form provides structure for consistency and transparency in reporting. For further information on Nature Portfolio policies, see our Editorial Policies and the Editorial Policy Checklist.

Please do not complete any field with "not applicable" or n/a. Refer to the help text for what text to use if an item is not relevant to your study.
For final submission: please carefully check your responses for accuracy; you will not be able to make changes later.

Statistics

For all statistical analyses, confirm that the following items are present in the figure legend, table legend, main text, or Methods section.

n/a	Confirmed	
☐	☒	The exact sample size (*n*) for each experimental group/condition, given as a discrete number and unit of measurement
☐	☒	A statement on whether measurements were taken from distinct samples or whether the same sample was measured repeatedly
☐	☒	The statistical test(s) used AND whether they are one- or two-sided<br>*Only common tests should be described solely by name; describe more complex techniques in the Methods section.*
☐	☒	A description of all covariates tested
☐	☒	A description of any assumptions or corrections, such as tests of normality and adjustment for multiple comparisons
☐	☒	A full description of the statistical parameters including central tendency (e.g. means) or other basic estimates (e.g. regression coefficient) AND variation (e.g. standard deviation) or associated estimates of uncertainty (e.g. confidence intervals)
☐	☒	For null hypothesis testing, the test statistic (e.g. *F*, *t*, *r*) with confidence intervals, effect sizes, degrees of freedom and *P* value noted<br>*Give P values as exact values whenever suitable.*
☒	☐	For Bayesian analysis, information on the choice of priors and Markov chain Monte Carlo settings
☐	☒	For hierarchical and complex designs, identification of the appropriate level for tests and full reporting of outcomes
☒	☐	Estimates of effect sizes (e.g. Cohen's *d*, Pearson's *r*), indicating how they were calculated

Our web collection on statistics for biologists contains articles on many of the points above.

Software and code

Policy information about availability of computer code

All tissue samples were immediately snap-frozen in liquid nitrogen post-collection and stored at -80°C. DNA and RNA were extracted from bulk frozen tissues using an AllPrep DNA/RNA Kit (Qiagen), and from blood samples using a Nucleon Genomic Extraction kit (Gen-Probe), following the manufacturer's instructions. DNA and RNA quantification and quality control were conducted using both Nanodrop and High Sensitivity Qubit (Thermo Fisher Scientific).
Hematoxylin and eosin-stained frozen sections with histologically confirmed IPMN or PDAC samples, as determined by an expert pathologist, and with an adequate amount of DNA and RNA, were deemed suitable for sequencing. All H/E slides were digitised for a second review by an expert pathologist and for cellular quantification.

Data collection

Data analysis

Read alignment and somatic variant calling
Lanes of paired raw FASTQ reads from each sample were merged through fastqtobam tool and then aligned to human reference genome (hg38) using Sanger bwamem2 mapping workflow cgpWGS330 (ftp://ftp.sanger.ac.uk/pub/cancer/dockstore/human/GRCh38_hla_decoy_ebv/core_ref_GRCh38_hla_decoy_ebv.tar.gz).
Somatic SNVs and InDels were identified from matched normal and tumour pairs using Sanger cgpwgs210 within a singularity (https://github.com/cancerit/dockstore-cgpwgs/wiki/ Running-under-singularity) and Mutect2 from GATK 4.1.8 (https://gatk.broadinstitute.org/hc/en-us/sections/ 360009656231-4-1-8-0). Driver genes of interest were identified as missense and nonsense SNVs, and as frameshift deletions / insertions and essential splice site deletions.
In order to generate VCF datasets for further analysis, mutational variants from these tools were selected with pass parameter and finally intersected using bcftools 1.11. In parallel, Breakpoint variants were isolated and assembled by cgpwgs210-BARSS. The SV files across all samples were filtered via SURVIVOR software and processed per sample using Python package Scikit-Allel to compute aggregated counts of each SV type: deletion, inversion, translocation, tandem duplication and multiple features. Fifty known drivers were queried from the final relevant datasets based on COSMIC cancer gene census.
Mutation and copy number signature profiling
Mutation signatures were analysed by using SigProfilerExtractor based on a non-negative matrix factorization (NNMF) framework (v0.0.5.77; https://github.com/AlexandrovLab/ SigProfilerExtractor). Signatures from De novo extraction and decomposition were profiled as single-base substitution (SBS96), double-base substitution (DBS78) and small insertion-deletion (ID83). Using ASCAT matrix generated from CNA datasets, copy number signatures were extracted by SigProfilerExtractor. Based on the updated known signatures as in COSMIC database, SigProflerAssignment was utilised to retrieve decomposed signatures.
In order to minimise NNMF artefact potentials, initial signature extraction was performed with all available samples simultaneously[8] and each single signature assignment per sample was finally determined by the probability matrix.

Mutation clustering and phylogenetic tree reconstruction
To model subclonal structure and construct phylogenetic tree from multiple normal-tumour matched samples, we combined various genomic datasets and applied a number of bioinformatics tools. For each sample, mutation allele fractions (MAF) of SNV and Indel were prepared by using alleleCounter and vafCorrect. Together with copy number and cellularity, these outcomes were submitted for clustering mutations to their mutation copy number using a previously described Bayesian Dirichlet process (DPClustering), (v2.2.8; https://github.com/Wedge-lab/dpclust/releases). This estimation per sample was extended into n dimensions for 12 IPMN-PDAC cohorts with n related samples, where the numbers of mutant reads obtained from multiple related samples were modelled as independent binomial distributions. Variants from each cohort with an upper CCF boundary above and below 1 were considered to be clonal and subclonal respectively.
Annotation of the trees with mutations and signatures
To annotate each tree with oncogenic or putative oncogenic alterations, in-house programs were utilised to connect multiple genomic variants and identify specific significance, including SNV, Indels, SV, CNV, mutation signature and cluster assignment information from mutation caller, Batterberg, signature extractor and msDPClustering.
RNAseq analysis
Sequencing quality of fastq files was assessed with FastQC (https:// github.com/s-andrews/FastQC) (v 0.11.9) and files were processed with fastp (v 0.21.0) using default settings. Quantification was performed against GRCh38 using Salmon (v 1.4.0). Salmon quantification results were imported into a DESeqDataSet using DESeq2 (v 1.38.3). Transcripts were mapped to genes using EnsDb.Hsapiens.v86 (v 2.99.0). Read count data were filtered to retain only those with normalised counts >= 5 in at least 18 samples (18,409 genes retained). Reads were transformed using the DESeq2 'vst' function. PCA was performed with plotPCA (DESeq2).
Scores were calculated for 50 Hallmark gene sets from MSigDB. Scores were calculated using the singscore package and the VST transformed counts.
Cell type proportions were estimated using the EPIC method as implemented in the immunedeconv package using TPM counts. The ESTIMATE method was used to calculate stromal, immune and overall ESTIMATE scores, implemented in the estimate (https://bioinformatics.mdanderson.org/ estimate/rpackage.html) package.
Data Processing and Statistical Analysis
Pipeline running, data processing, statistical analysis and visualisation were mainly performed in R and Python programming languages based on High Performance Computing (HPC) cluster, The University of Manchester. To determine an association between lesion grade and the presence of mutational types, we applied Fisher's Exact Test for genomic alterations and the Mann–Whitney Test for expression and deconvolution data across different tumor stages. To assess differences in cell count data between Clusters 1 and 2, median values from samples of each case were considered, while for differential gene expression between transcriptomic clusters, two-sided Student's *t*-tests were used. Additionally, to compare the overall number of SNVs, indels, SVs, and TMB between IPMN and PDAC, we used the Fisher–Pitman Permutation Test. A p value of less than 0.05 was considered statistically significant. Multiple hypothesis testing was controlled using the Benjamini–Hochberg procedure, with FDR < 0.05 considered statistically significant. Specifically, we applied Fisher–Pitman permutation tests to compare the number of SNVs, indels, and structural variants (SVs) across tumor types and genomic regions; Fisher's exact tests to compare the frequency of driver mutations (including SNVs, indels, SVs, and CNAs) and SV signatures across tumor types; and permutation-based Wilcoxon tests for pairwise comparisons of timing group proportions. To determine whether specific cancer driver mutations are preferentially clonal early or subclonal, we applied a bootstrap resampling approach to estimate odds ratios (ORs) between subclonal and early clonal occurrences across tumors. For each driver, we computed the median OR and the 95% confidence interval (CI), defined as the 2.5th to 97.5th percentiles of the bootstrap distribution. A driver was considered significantly enriched in subclonal or clonal early mutations if the 95% CI of the OR did not include 1. Specifically, a CI greater than 1 indicated significant enrichment in subclonal timing, a CI less than 1 indicated significant enrichment in early clonal timing, and a CI that overlapped 1 indicated no significant enrichment.

For manuscripts utilizing custom algorithms or software that are central to the research but not yet described in published literature, software must be made available to editors and reviewers. We strongly encourage code deposition in a community repository (e.g. GitHub). See the Nature Portfolio guidelines for submitting code & software for further information.

Data

Policy information about availability of data
All manuscripts must include a data availability statement. This statement should provide the following information, where applicable:
- Accession codes, unique identifiers, or web links for publicly available datasets
- A description of any restrictions on data availability

April 2023

- For clinical datasets or third party data, please ensure that the statement adheres to our policy

Whole-genome sequencing (WGS) and RNA-seq raw data have been deposited in the European Genome-phenome Archive (EGA) under accession number EGAS50000001182 (https://submission.ega-archive.org/studies/EGAS50000001182). Processed datasets supporting the findings of this study are available at the following repositories:

- https://github.com/Wedge-lab/IPMNPDACpaperArchive/tree/main/IPMNPDAC_WGS/Data
- https://github.com/Wedge-lab/IPMNPDACpaperArchive/tree/main/IPMNPDAC_WGS/Data/sigDPC
- https://github.com/Wedge-lab/IPMNPDACpaperArchive/tree/main/IPMNPDAC_WGS/Data/svDriverCluster
- https://github.com/Wedge-lab/IPMNPDACpaperArchive/tree/main/IPMNPDAC_WGS/Data/cnvDriverCluster

Research involving human participants, their data, or biological material

Policy information about studies with human participants or human data. See also policy information about sex, gender (identity/presentation), and sexual orientation and race, ethnicity and racism.

Reporting on sex and gender	This study has no sex and/or gender-based analyses. Gender data for each patient are detailed in the sample manifest
Reporting on race, ethnicity, or other socially relevant groupings	No data about race, ethnicity, or other socially relevant groupings was collected for this study
Population characteristics	The IPMN cohort consisted of A cohort of 12 patients underwent pancreatic resection for high-risk IPMNs following clinical guidelines (Table S1). Forty-seven tumor samples, plus matching normal tissue from each patient, were harvested from 12 surgical specimens (mean of 4 regions per tumor, range 2–6). Whole-genome sequencing (WGS) was successfully performed on 54 samples. Thirty-seven tumor samples were included in the clonal analysis, excluding 5 with low tumor purity or mutation counts. Transcriptome sequencing (RNAseq; average 100 million paired reads) was performed on 36 tumor samples, 32 of which had matching WGS data.
Recruitment	Participants were selected based on clinical and radiological data indicative of IPMN with a high risk for cancer, without consideration of factors such as age or gender, ensuring an unbiased selection process. Sample collection was conducted during gross examination, with validation performed through pathological analysis of H&E-stained slides from fresh-frozen tissue and corresponding diagnostic FFPE slides.
Ethics oversight	The materials used have been collected under Ethics Committee Approval (ECA) of both institutions (Glasgow: ref. 22/WS/0020; Verona: program 1885 protocol 52438 and amended with protocol 25982). All participants provided written informed consent for sample collection and subsequent analyses publication.

Note that full information on the approval of the study protocol must also be provided in the manuscript.

Field-specific reporting

Please select the one below that is the best fit for your research. If you are not sure, read the appropriate sections before making your selection.

[X] Life sciences [ ] Behavioural & social sciences [ ] Ecological, evolutionary & environmental sciences

For a reference copy of the document with all sections, see nature.com/documents/nr-reporting-summary-flat.pdf

Life sciences study design

All studies must disclose on these points even when the disclosure is negative.

Sample size	IPMN are rare tumors so we tried to collect as many samples as possible for each case. Our multiregional sampling protocol was designed to collect samples that spanned all grades of dysplasia and invasive cancer within the same neoplastic lesion. Each pancreatic lesion was divided into 4 to 9 segments, with the relative positions of each segment meticulously recorded in a clockwise numeric order for spatial reconstruction, adhering to the institutional grossing protocol. During the grossing examination, care was taken to ensure that the collected samples were from segments of the same lesion with no normal tissue in between, to avoid the inclusion of concomitant but separate tumours. Blood, duodenum, or spleen samples were utilized as germline references.
Data exclusions	All samples included in this study underwent rigorous pathological examination. Samples with no neoplastic tissue on H&E pathological assessment were excluded. One sample with only stromal component (case 13_S4, chronic pancreatitis) was included due to the adjacency of neoplastic tissue. Samples failing QC or WGS were excluded from further analysis. Cases with fewer than two successfully sequenced samples per case, the minimum required to perform clonal analysis, were excluded from the study.
Replication	Information were included in the figure legends.
Randomization	Randomization was not applicable due to the purpose of the study
Blinding	Whole-genome and RNA sequencing analyses were performed independently of histopathological evaluations. Pathological assessment of fresh frozen tissue samples and FFPE diagnostic slides was conducted by expert pancreatic cancer and IPMN pathologists, who were blinded to the molecular findings.

Reporting for specific materials, systems and methods

We require information from authors about some types of materials, experimental systems and methods used in many studies. Here, indicate whether each material, system or method listed is relevant to your study. If you are not sure if a list item applies to your research, read the appropriate section before selecting a response.

Materials & experimental systems

n/a	Involved in the study
☐	☒ Antibodies
☐	☐ Eukaryotic cell lines
☐	☐ Palaeontology and archaeology
☐	☐ Animals and other organisms
☐	☐ Clinical data
☐	☐ Dual use research of concern
☐	☐ Plants

Methods

n/a	Involved in the study
☐	☐ ChIP-seq
☐	☐ Flow cytometry
☐	☐ MRI-based neuroimaging

Antibodies

Antibodies used

For all cases, the following antibodies were tested: P53 (clone: DO-7; 1:50 dilution; Novocastra/UK), SMAD4 (B-8; 1:1000; Santa Cruz/USA) and, for mismatch repair (MMR) proteins, MLH1 (clone: ES05; dilution 1:30; Dako / Germany), PMS2 (MRQ-28, 1:150, Cell Marque Impath Menarini / Italy), MSH2 (FE11; 1:30, Dako), and MSH6 (EP49; 1:100, Dako).

Validation

Specific immunohistochemical staining on FFPE diagnostic slides of non-invasive IPMNs and corresponding PDAC was performed using standardized institutional protocols, as detailed in the Methods section, and evaluated according to the manufacturers' instructions.

This checklist template is licensed under a Creative Commons Attribution 4.0 International License, which permits use, sharing, adaptation, distribution and reproduction in any medium or format, as long as you give appropriate credit to the original author(s) and the source, provide a link to the Creative Commons license, and indicate if changes were made. The images or other third party material in this article are included in the article's Creative Commons license, unless indicated otherwise in a credit line to the material. If material is not included in the article's Creative Commons license and your intended use is not permitted by statutory regulation or exceeds the permitted use, you will need to obtain permission directly from the copyright holder. To view a copy of this license, visit http://creativecommons.org/licenses/by/4.0/
